# Transcription Analysis of Liver and Muscle Tissues from Landrace Finishing Pigs with Different Feed Conversion Ratios

**DOI:** 10.3390/genes13112067

**Published:** 2022-11-08

**Authors:** Zhixin Wang, Yingzhi He, Zhen Tan

**Affiliations:** School of Animal Science and Technology, Hainan University, Haikou 570228, China

**Keywords:** pig, liver, muscle, feed efficiency, RNA-Seq

## Abstract

The efficiency of feed utilization determines the cost and economic benefits of pig production. In the present study, two pairs of full-sibling and two pairs of half-sibling female Landrace finishing pigs were selected, with each pair including individuals with different feed conversion rates, with liver and longissimus muscle tissue samples collected from each group for transcriptome analysis. A total of 561 differentially expressed genes (DEGs), among which 224 were up-regulated and 337 were down-regulated, were detected in the liver transcriptomes in the high-feed efficiency group compared to the low-feed efficiency group. The DEGs related to phosphorus and phosphate metabolism, arginine biosynthesis, chemical carcinogenesis, cytokine-cytokine receptor interaction, the biosynthesis of amino acids, and drug metabolism-cytochrome P450 in liver tissue were also associated with feed efficiency. In total, 215 DEGs were screened in the longissimus muscle tissue and were mainly related to disease and immune regulation, including complement and coagulation cascades, systemic lupus erythematosus, and prion diseases. The combination of gene expression and functional annotation results led to the identification of candidate feed efficiency-related biomarkers, such as *ARG1*, *ARG2*, *GOT1*, *GPT2*, *ACAA2*, *ACADM*, and *ANGPTL4*, members of cytochrome P450 family, and complement component family genes. Although the novel feed efficiency-related candidate genes need to be further evaluated by a larger sample size and functional studies, the present study identifies novel candidate biomarkers for the identification of functional SNPs underlying porcine feed efficiency.

## 1. Introduction

In modern livestock production, feed accounts for 60% of the total cost [1]. Reducing feed costs is of great significance to the pig industry; feed efficiency (FE) affects production costs and offers economic benefits, thus, improving feed efficiency is important in pig production.

In addition, improving FE can reduce feed nutrient wastage, waste-related environmental pollution, and the current severe breeding pollution [1]. Like other traits, FE is determined by both genetics and environmental factors, with genetics accounting for 30% of individual FE variants [2,3]. The two metrics most commonly used to evaluate FE are residual feed intake (RFI) and feed conversion ratio (FCR) [1]. The heritability of the FCR is 0.13–0.31, while RFI is 0.14–0.40 [4], and the correlation between the FCR and RFI is positive and high (R equals 0.76–0.99), with both low RFI and FCR values corresponding to high FE [4].

Several important quantitative trait loci (QTL) regions and candidate genes were identified to be associated with FE traits by genome-wide association studies (GWAS) in pigs [5,6,7,8]. However, putative FE genes participated in a wide range of biological processes [5,6] and in many functional pathways in different tissues through gene expression profiles [9], suggesting that the biological strategies used to improve FE are diverse. Tissue- and organ-specific transcriptomes and miRNA profiles have been widely used to elucidate the molecular basis of inter-individual differences in FE [10,11,12,13]. In pigs, FE-related transcriptome research has focused on energy homeostasis and energy demand [9,10,14,15,16], mainly in the liver and adipose tissue [17] and in intestinal, blood, and muscle tissue [13,18,19]. The liver is the most important organ involved in metabolism and, as such, plays an important role in converting newly acquired energy into the muscle or adipose tissue, thereby affecting FE [20,21]. The analysis of liver transcriptomes from pigs with different FE phenotypes indicates that FE is associated with a variety of biological processes, including cell proliferation, vitamin A metabolism, protein synthesis and catabolism, lipid metabolism, carbohydrate metabolism, mitochondrial activity, and glucose synthesis, as well as linked signal transduction (e.g., oxidative stress, inflammation, and immune response) [7,9,11,16,19,22]. Aside from the liver, muscles also contain the key metabolically relevant tissues for EF [23]; the majority of muscle tissue proteins (73%) are related to FE-related biological processes, including energy metabolism (28%), muscle contraction (12%), conversion (12%), cellular amino acid-derived metabolic processes (11%), and glucose metabolism (10%). These studies have established a basis for investigating the physiological roles of the liver and muscle tissue in regulating FE in pigs. In addition to tissue specificity, the transcriptome results of different breeds of pigs at different growing periods were also quite different [9,12,24].

Accordingly, this study aimed to use transcriptome sequencing to identify differentially expressed genes (DEGs) in the liver and longissimus muscle tissues of high-FE and low-FE groups. The functional annotation of DEGs helps determine whether they are associated with growth traits and/or microbial digestion. Therefore, the present study may provide insight into the role of hepatic transcriptome architecture in pig liver function and, thus, in the development of high-FE pigs.

## 2. Materials & Methods

### 2.1. Animals, Phenotype Selection, and Sample Collection

A total of 120 purebred Landrace female pigs were obtained from the Tianjin Ninghe primary pig breeding farm (Ninghe, China). All experimental pigs were weaned at the age of 28 days and raised under the same commercial formula diet, mainly composed of corn, soybean meal, lysine, and calcium hydrogen phosphate, and the same controlled farm management conditions. From 120 to 165 d, their body weights ranged from 50 to 90 kg; pigs with divergent FCRs groups (20 highest and 20 lowest FEs) were identified by the Velos automatic feeding system. In total, eight pigs, with two full-sibling pairs and two half-sibling pairs of female Landrace finishing pigs, selected from each of the two groups, with each pair including opposing FCR phenotypes, were used from our previously established pig population described by Wang et al. [25].

After being stunned with a captive bolt and exsanguinated, the selected pigs were slaughtered at 166 d old, and tissue samples from the front of the left liver and middle of the longissimus muscle were taken aseptically immediately afterward. The HL and HM group were defined as the liver and longissimus muscle tissues of pigs with high FE and low FCR values, while individuals with low FE and high FCR values were in the LL and LM groups, respectively (Supplementary Table S1). All samples were collected in sterile tubes and stored in liquid nitrogen until further analysis.

### 2.2. RNA Isolation

Total RNA was extracted from the liver and muscle tissue samples using the RNAsimple Total RNA Kit (Tiangen Biotech Co. Ltd., Beijing, China), following the manufacturer’s instructions. RNA degradation and contamination were evaluated using 1% agarose gel electrophoresis, and RNA purity and integrity were evaluated using a NanoPhotometer spectrophotometer (Implen, Munich, Germany) and Agilent 2100 Bioanalyzer (Agilent, Santa Clara, CA, USA), respectively.

### 2.3. RNA Library Preparation and Sequencing

Sequencing libraries were generated using 1 μg RNA from each sample and the NEBNext Ultra RNA Library Prep Kit(Lumiprobe Corporation, Baltimore, MA, USA) for Illumina following the manufacturer’s recommendations, with index codes added to attribute sequences to each sample. Briefly, mRNA was purified from total RNA using poly T oligo-attached magnetic beads and fragmented using divalent cations under elevated temperature in NEBNext First Strand Synthesis Reaction Buffer (5×). First-strand cDNA was then synthesized using random hexamer primers and M-MuLV Reverse Transcriptase (RNaseH-), and second-strand cDNA was synthesized using DNA Polymerase I and RNase H. Remaining overhangs were converted into blunt ends via exonuclease/polymerase activities, and after adenylation of the 3’ ends, the cDNAs were ligated to NEBNext Adaptor for hybridization. Library fragments of 250–300 bp in length were selected using the AMPure XP system (Beckman Coulter, Beverly, Massachusetts, USA) and then incubated with 3 μL USER Enzyme (NEB) at 37 °C for 15 min, followed by 5 min at 95 °C. PCR was then performed using Phusion High-Fidelity DNA polymerase, universal PCR primers, and index (X) primers, and the resulting products were purified (AMPure XP system). The qualities of the resulting libraries were evaluated using the Agilent Bioanalyzer 2100 system, and index-coded sequences corresponding to each sample were clustered using a cBot Cluster Generation System and a TruSeq PE Cluster Kit (v3-cBot-HS; Illumina, San Diego, CA, USA) according to the manufacturer’s instructions. Finally, the clustered library preparations were sequenced (150 bp paired-end reads) using an Illumina Hiseq 4000 platform.

### 2.4. Read Processing and Mapping

The raw RNA-Seq reads were processed using FASTQC [26] to remove reads with low quality (>50% of read length with base threshold quality score of <20) and reads containing adapter sequences or unknown (N) bases. The paired-end clean reads were then mapped to the indexed Sscrofa11.1 reference genome, which was downloaded from the Ensembl database using Hisat2 v2.0.5 [27], and the numbers of reads mapped to exons, introns, and intergenic positions were calculated using RSeQC (version 3.0.1) (Boston, MA, USA) [28].

### 2.5. Differential Gene Expression Analysis and qPCR Validation

The numbers of reads mapped to specific genes were calculated using the featureCounts function in Subread v1.5.0 [29], and the numbers of fragments per kilobase of transcript sequence per million base pairs sequenced (FPKM) were calculated for each gene based on gene length and corresponding read count [30]. Differential expression of the genes between groups was evaluated in R using the ‘DESeq2’ package (1.16.1) [26]. The resulting *p*-values were adjusted using Benjamini and Hochberg’s approach for controlling the false discovery rate, and genes with adjusted *p*-values of <0.05 were defined as differentially expressed.

The DEG assignments were then validated using qPCR. Briefly, RNA from each sample was converted into cDNA using TransScript First-Strand cDNA Synthesis SuperMix (TransGen Biotech, Beijing, China) following the manufacturer’s instructions and then qPCR amplified in 20-μL reactions using the SYBR Premix Ex Taq Kit (Takara Biotechnology, Shiga, Japan), gene-specific primers (Supplementary Table S2), and an ABI Step One Plus Real-Time PCR system (Applied Biosystems, Foster City, CA, USA). Relative mRNA abundance was calculated using the 2^−ΔΔCT^ method and normalized to the endogenous reference gene *GAPDH* [31].

### 2.6. DEGs Functional Annotation Clustering

The GO and pathway enrichment analysis of DEGs functional annotation was performed using DAVID v6.8 (http://david.abcc.ncifcrf.gov/20210701 accessed on 10 October 2022) [32,33] and was used to designate DEGs (*p* < 0.05) and perform both Gene Ontology (GO) enrichment and KEGG pathway analysis. GO terms with corrected *p*-values of <0.05 were considered significantly enriched DEGs. The Kyoto Encyclopedia of Genes and Genomes (KEGG) is a database resource used for investigating the high-level functions and utilities of biological systems from molecular-level information (http://www.genome.jp/kegg/20210701 accessed on 10 October 2022) [34]. The interaction network of proteins was developed using STRING (http://www.string-db.org/20210701 accessed on 10 October 2022), and ToppCluster was used to generate the potentially important network of pathways and DEGs, with a *p*-value cut-off value of 0.05 [35].

## 3. Results

### 3.1. RNA-Seq Data

A total of 16 cDNA libraries for liver and muscle tissues were constructed. Each liver sample yielded 39.98–52.38 million clean reads. Average unique mapped reads accounted for 92.67% of each sample, with more than 94% of these mapped reads included within coding sequences (CDSs). Meanwhile, each muscle tissue sample yielded 41.52–49.55 million clean reads. Average unique mapped reads accounted for 92.37% of each sample (Supplementary Table S3).

### 3.2. Gene Expression Analysis

The expression levels of all the genes in the eight liver tissues were between 13,221 and 17,766, with more than 17,600 common genes in both groups. The high reproducibility for the genes expressed between the two groups indicated that the majority of the liver transcriptome appears to be conserved between them (Supplementary Figure S1). A number of genes were highly expressed in the liver tissues of both groups, including Cytochrome C oxidase subunit 1, 2, and 3 (*COX1*, *COX2*, and *COX3*), ATP synthase F0 subunit 6 (*ATP6*), Cytochrome b (*CYTB*), apolipoprotein E (*APOE*), NADH-ubiquinone oxidoreductase chain 1, 2, 3, 4, 4L, 5, and 6 (*ND1*, *ND2*, *ND3*, *ND4*, *ND4L*, *ND5*, and *ND6*), apolipoprotein C3 (*APOC3*), haptoglobin (*HP*), and albumin (*ALB*). These were the top 16 highly expressed genes in both groups and thus could play important roles in the liver tissue.

In muscle tissue samples, the number of expressed genes was from 16,805 to 17,788, where the highest expressed genes in both groups were actin alpha 1 (*ACTA1*), aldolase, fructose-bisphosphate A (*ALDOA*), glyceraldehyde-3-phosphate dehydrogenase (*GAPDH*), creatine kinase, M-type (*CKM*), *COX1* and *COX3*, myosin light chain phosphorylatable, light chain 1, heavy chain 4 (*MYLPF*, *MYL1*, and *MYH4*), troponin T3, troponin I2 (*TNNT3* and *TNNI2*), tropomyosin 1 (alpha) and tropomyosin 2 (beta) (*TPM1* and *TPM2*), enolase 3 (*ENO3*), glycogen phosphorylase (*PYGM*), pyruvate kinase (*PKM*), and ATP synthase F0 subunit 6, ATPase sarcoplasmic/endoplasmic reticulum Ca^2+^ transporting 1 (*ATP6* and *ATP2A1*).

**Figure 1:**
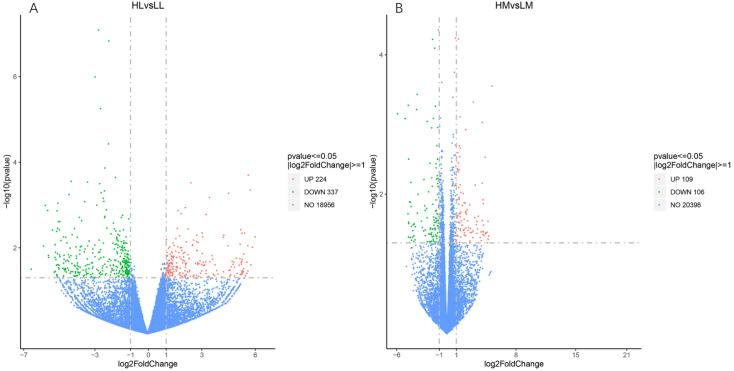
Volcano plot of the genes differentially expressed between HL vs. LL (**A**) and HM vs. LM (**B**) groups. The green dots represent the significantly over-expressed genes in the HL/HM group; the red dots represent the significantly under-expressed genes in HL/HM group; and the blue dots represent the genes whose expression levels did not reach statistical significance.

A comparison of the liver and longissimus muscle transcriptomes of high- and low-FE groups revealed 561 and 215 DEGs, respectively, with criteria of |log2FoldChange| > 1, a *p*-value of less than 0.05, and expressed in paired comparison groups, including 224 up-regulated and 337 down-regulated DEGs (HL vs. LL) and 109 up-regulated and 106 down-regulated DEGs (HM vs. LM) (Figure 1). However, with an adjusted *p*-value less than 0.05, only four genes were detected in liver tissues, cytochrome P450, family 1, subfamily A, polypeptide 2 (*CYP1A2*), acetyl-CoA acyltransferase 2 (*ACAA2*), immunoglobulin heavy constant alpha 2-like (*LOC102165485*), and tripartite motif containing 26 (*TRIM26*). No significant DEGs were identified among the longissimus muscle tissue transcriptomes.

### 3.3. Functional Annotation Clustering of DEGs

**Figure 2:**
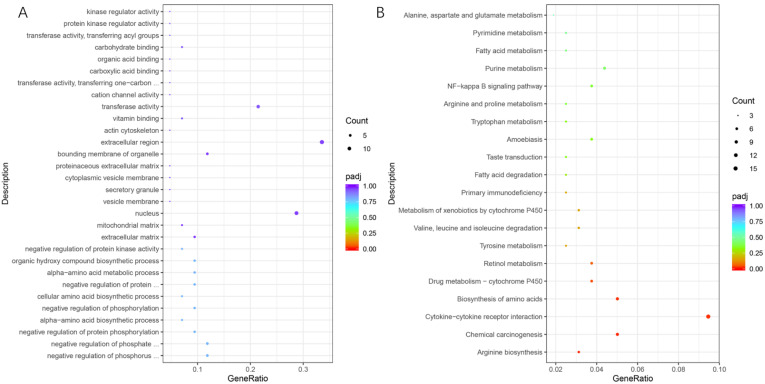
Enrichment of gene ontology (GO) terms (**A**) and KEGG pathways (**B**) in HL vs. LL. HL, liver samples of the high-EF group; LL, liver samples of the low-EF group.

DEGs were annotated by using DAVID Bioinformatics Resources 6.8 to explore the major GO terms and KEGG pathways. In liver tissues, with a *p*-value less than 0.05 as the standard, biological process GO analysis identified 31 significantly enriched GO terms, including the negative regulation of the phosphorus metabolic process, the phosphate metabolic process, of protein phosphorylation, the alpha-amino acid biosynthetic process, phosphorylation, the cellular amino acid biosynthetic process, et al. Molecular functional GO analysis identified two significantly enriched GO terms, vitamin binding and transferase activity (Figure 2A). However, no GO functional terms were found to be enriched when using a more stringent Benjamini-adjusted *p*-value (q < 0.05). For KEGG enrichment analysis, the DEGs were significantly enriched in 19 pathways (*p*-value < 0.05) (Figure 2B). With the Benjamini adjusted *p*-value (q < 0.05), the significant enrichment pathways of annotated DEGs were arginine biosynthesis, chemical carcinogenesis, cytokine-cytokine receptor interaction, biosynthesis of amino acids, and drug metabolism-cytochrome P450.

**Figure 3:**
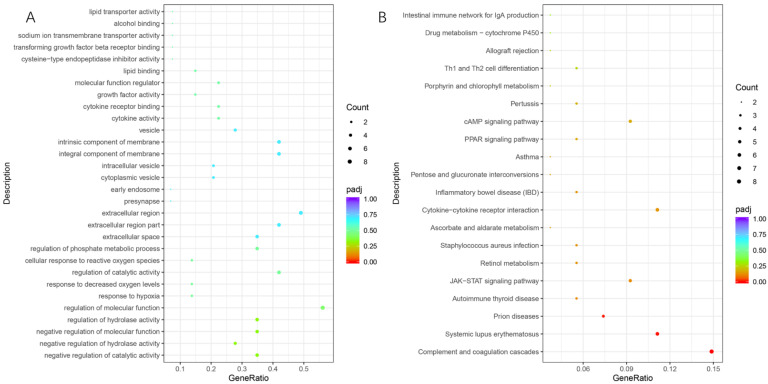
Enrichment of GO terms (**A**) and KEGG pathways (**B**) in HM vs. LM. HM, longissimus muscle samples of high-EF group; LM, longissimus muscle samples of low-EF group.

In longissimus muscle tissues, 45 biological process GO terms were identified, including negative regulation of catalytic activity, hydrolase activity, molecular function, and regulation of hydrolase activity, molecular function, et al. Extracellular space was only one significant GO term of cellular component, and molecular functional GO analysis identified two significantly enriched GO terms, cytokine activity, and cytokine receptor binding (Figure 3A). With a *p*-value less than 0.05, the DEGs were significantly enriched in 20 pathways (Figure 3B). The pathways related to complement and coagulation cascades, systemic lupus erythematosus, and prion diseases were significantly enriched using Benjamini-adjusted *p*-value (q < 0.05).

Protein interaction networks were constructed using STRING. DEGs were input into STRING, and discrete genes that were not associated with others in the network were removed. *CYP1A2* and *ACAA2* were found in the liver and more than 200 DEGs in the protein interaction network and were connected (Supplementary Figure S2A). In muscle tissue, complement component 4 (C4), C5, C6, C9, C8A, complement component 4 binding protein alpha (C4BPA), and angiopoietin-like 4 (*ANGPTL4*) were interconnected with other genes in the protein interaction network (Supplementary Figure S2B).

**Figure 4:**
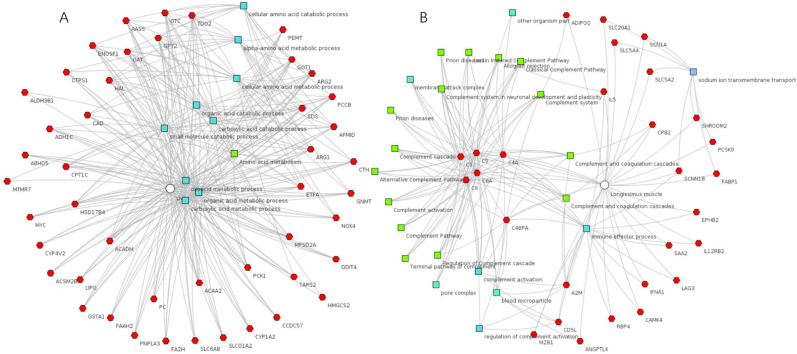
Relationships between differentially expressed genes, KEGG pathways, and GO terms of HL vs. LL (**A**) and HM vs. LM (**B**). Functional association analysis was performed using ToppCluster. Red hexagons: differentially expressed gene; green squares: pathway; blue squares: biological process; and gray squares: molecular function.

The DEG functional network was observed using ToppCluster software for each comparison (Figure 4). GO terms related to multiple acid catabolic processes and metabolic processes were detected and pathways related to amino acid metabolism were enriched in the HL vs. LL group, which is consistent with previous results obtained through DAVID. DGEs related to small molecules and the cellular catabolic process were also identified (Figure 4A). In longissimus muscle tissues, DEGs encoded proteins related to the activation cascade, blood microparticles, regulation of complement activation, immune effector process, and sodium ion transmembrane transport were detected (Figure 4B). Complement family genes, including C4, C5, C6, C9, C8A, and C4BPA, were associated with multiple metabolic pathways, forming a network.

### 3.4. Validation of DEGs Using qPCR

The regulation quantification of six DEGs (*ACAA2*, *TRIM26*, *ANGPTL4*, *CTGF*, *HP*, and *APOE*) including down-regulated, up-regulated, and high expression abundance genes, were selected for qPCR. For all six DEGs, the direction of gene expression changes was similar to those measured by qPCR and RNA-seq analysis (Supplementary Figure S3).

## 4. Discussion

Improving FE is one of the most efficient ways to increase the economic benefits of pig production. Therefore, it is important to investigate the mechanisms of FE in pig breeding to reduce costs and improve production efficiency. In the present study, the transcription profiles of the liver and longissimus muscle tissues from two pairs of full-sibling female Landrace finishing pigs and two pairs of half-sibling individuals, with each pair including both high- and low-FCR individuals, were systematically analyzed. Using closely related individuals with distinct phenotypes can reduce the noise caused by genetic background and, thus, reduce the occurrence of false-positive results [35,36,37].

Pigs mainly regulate feed utilization efficiency through the digestion and utilization of a large number of nutrients, and several of these molecular reaction mechanisms (e.g., lipid metabolism, enzyme activity, and related gene expression) are directly involved with liver and muscle tissues [38,39,40]. Genes associated with energy metabolism and immunity are considered key influencers of feed efficiency. The present study partially supports this hypothesis and provides novel findings of many potential candidate functional genes associated with FE in Landrace pigs. Although the genes with the highest expression in the liver were related to energy metabolism, these genes were not significantly different between the two groups. An analysis of breast muscle transcriptomes from FE-divergent native chickens resulted in the identification of DEGs related to oxidative phosphorylation (e.g., *COX1*, *COX2*, *COX3*, *ATP6*, *ND1*, *ND2*, *ND3*, *ND4*, *ND4L*, *ND5*, and *CYTB*) that were upregulated in high-FE chickens [41]. The most highly expressed genes in both the high- and low-FE groups were involved in tryptophan metabolism in this trial.

The DEGs identified among the liver tissues of high- and low-FE pigs were mainly related to phosphorus and phosphate metabolism and the amino acid biosynthetic process of GO analysis terms. Based on the KEGG analysis, three of the five different pathways related to immunity, cytokine-cytokine receptor interaction, chemical carcinogenesis, and drug metabolism-cytochrome P450 were significantly different, the DEGs including the C-C motif chemokine ligand family (*CCL4* and *CCL26*), C-C motif chemokine receptor family (*CCR6* and *CCR10*), interleukin family (*IL18RAP*, *IL12RB2*, *IL15RA*, and *IL17B*), and cytochrome P450 family (*CYP1A2*, *CYP2C49*, *CYP2C32*, and *CYP2C33*). Cytochrome P450 family genes were also identified as potential candidate genes in our previous study, through the screening of differentially expressed genes in intestinal mucosal tissues, indicating that these genes may be associated with FE traits in multiple tissues of pigs. Two of these traits belong to metabolism (arginine biosynthesis and biosynthesis of amino acids), and include the enriched DEGs such as the arginase family (*ARG1* and *ARG2*), glutamic-oxaloacetic transaminase 1 (*GOT1*), and glutamic-pyruvic transaminase 2 (*GPT2*). Energy-related metabolism pathways were also enriched, yet did not have a significant *p*-adjusted value. In addition, genes such as acetyl-CoA acyltransferase 2 (*ACAA2*) and acyl-CoA dehydrogenase medium chain (*ACADM*) are also related to fatty acid beta-oxidation.

Previous analysis of liver transcriptomes from castrated Yorkshire boars with high- and low-FCRs revealed DEGs that were primarily enriched in vitamin A, fatty acid, and steroid hormone metabolism [16]. For example, *CYP1A1* was involved in both vitamin A and steroid hormone metabolism. Both *CYP1A1* and *CYP1A2* belong to the cytochrome P450 family 1 subfamily A and participate in the metabolism of vitamins and fats. Moreover, members of the cytochrome P450 (*CYP*) superfamily of enzymes, which catalyze reactions involved in cholesterol and steroid synthesis, are down-regulated in the livers of high-FE pigs and cattle [9,42].

Participating in fatty acid elongation and degradation by catalyzing the last step of the mitochondrial β-oxidation spiral, *ACAA2* encodes an enzyme in the thiolase family that is involved in cell apoptosis and promotes cell proliferation and fatty acid metabolism [43]. Previous studies have reported that *ACAA2* overexpression can inhibit triglyceride production and cell proliferation and can induce apoptosis; in addition, the up-regulation of *ACAA2* can induce fatty acid prolongation [44].

The present study also determined that the high-FE group exhibited higher GTPase activity than the low-FE group. *GTP* is an important component of riboflavin (vitamin B2), which is synthesized from *GTP* and ribose 5-phosphate. As a substrate [45], *GTP* also participates in a variety of other biological activities (e.g., proliferation, differentiation, adhesion, and intracellular transport) which promote physiological growth and development and improve FE [46]. Based on the networks of DEGs, gene ontology, and KEGG pathways, functional genes, such as solute carrier (*SLC*) gene families which contain many glucose transporters, were enriched and considered tumor suppressors [47].

Muscle tissue transcriptome analysis revealed enrichment in terms related to disease and immune regulation, such as “blood coagulation response to the virus and negative regulation of apoptotic processes”. One potential explanation is that, in low-FE individuals that have more nutrients, gene expression is mainly related to material metabolism, whereas, in high-FE individuals, gene expression is mainly related to the steady-state activities of maintaining healthy levels or defending against severe environmental challenges. Both our previous study and another previous study on intestinal mucosa transcriptomes revealed that most DEGs were related to immunity and diseases, regardless of the FE group [18,35].

The protein-coding gene *ANGPTL4* was identified as an important candidate gene by the protein interaction network. *ANGPTL4* is a secreted glycoprotein that plays a physiological role in lipid metabolism and is mainly expressed in the liver and adipose tissue. It also inhibits the activity of LPL, thereby promoting elevated triglyceride levels [48,49] and, thus, improving FE. Based on networks of DEGs, gene ontology, and KEGG pathways, complement component 4 (*C4*), *C5*, *C6*, *C9*, *C8A*, complement component 4 binding protein alpha (*C4BPA*), and angiopoietin-like 4 (*ANGPTL4*) were interconnected with other genes in the protein interaction network. Complementary component-related genes are known for playing an important role in the classical pathway of complement systems and body homeostasis [50].

Annotated DEGs in the liver and longissimus muscle tissues of finishing female Landrace pigs with high and low FCR values were compared. A number of potential functional candidate genes were screened through transcriptomic sequencing analysis. Some DEGs were consistent with previous studies and several novel genes were found, further indicating that the complexity of feed efficiency traits and the ever-improving mining of genomics information are needed for the in-depth analysis of this trait. Although this study used siblings or half-sibling individuals to eliminate the influence of genetic background as much as possible, the small sample size is a major shortcoming of this study and needs to be expanded in future studies.

## 5. Conclusions

The present study compared the liver and longissimus muscle tissue transcriptomes of high- and low-FE finishing female Landrace pigs. The functional analysis of DEGs suggested that genes related to phosphorus and phosphate metabolism, arginine biosynthesis, amino acid biosynthesis, and immunity in liver tissue were associated with feed efficiency. In the longissimus muscle tissues, DEGs were mainly related to disease and immune regulation. In addition to many DEGs being consistent with previous studies, several novel genes were also detected, further indicating that tissue-specific pathways contribute to differences in feed efficiency and the complexity of feed efficiency traits. The genes, *ARG1*, *ARG2*, *GOT1*, *GPT2*, *ACAA2*, *ACADM*, *ANGPTL4*, members of the cytochrome P450 family, and complement component family genes are promising as potential candidate biomarkers for the identification of functional SNPs.

## Data Availability

The deep-sequenced RNA-Seq data for the eight female Landrace pigs are available from the NCBI Sequences Read Archive, under Bioproject: PRJNA234336 and Bioproject: PRJNA287472.

## Supplementary Materials

The following supporting information can be downloaded at: https://www.mdpi.com/article/10.3390/genes13112067/s1, Figure S1: Gene expression levels in liver tissue for female landrace pigs with high and low feed efficiency groups. (A) Venn diagrams show the total number of expressed genes in the two groups. (B) The gene expression counts in HL and LL groups after FPKM quantification, respectively. Figure S2: Prediction of protein-protein interactions of DEGs using STRING in HL vs. LL (A) and HM vs. LM (B). (A) Up-regulated DEGs, (B) Down-regulated DEGs. Figure S3: Validation of RNA Sequencing results by qPCR of liver samples (A) and longissimus muscle tissues samples (B). Table S1: Individuals selected for transcriptome. Table S2: Forward and reverse primers used for qPCR validation from RNA-Seq. Table S3: Transcriptome sequencing data of liver and longissimus muscle tissues of high and low feed efficiency.
