# Supplementary material for: Transcription Analysis of Liver and Muscle Tissues from Landrace Finishing Pigs with Different Feed Conversion Ratios
Source: Genes (Basel). 2022 Nov 8;13(11):2067. doi: 10.3390/genes13112067 (PMC9690258; doi:10.3390/genes13112067)
Supplement: Supplementary file 1 [file genes-13-02067-s001.zip › genes-2013343-SI/Supplementary Table S1.pdf]

Supplementary Table S1 Individuals selected for transcriptome.

Group	Sample	Initial BW (kg)	Final BW (kg)	DFI (kg)	ADG (kg)	FCR	Father ID	Mother ID
HL/HM	HL1/HM1	49.4	92.5	1.935	0.958	2.02	LLDA27813008791	LLNHPF713043113
	HL2/HM2	50.5	93.9	1.919	0.964	1.99	LLNHPF711011101	LLNHPF712131314
	HL3/HM3	50.2	92.5	1.908	0.940	2.03	LLDA27813008791	LLNHPF712304013
	HL4/HM4	51.5	91.6	1.978	0.891	2.22	LLNHPF712246400	LLNHPF714116103
LL/LM	LL1/LM1	50.6	81.8	2.156	0.693	3.11	LLDA27813008791	LLNHPF714105513
	LL2/LM2	50.1	83.2	2.126	0.736	2.89	LLNHPF711011101	LLNHPF713012411
	LL3/LM3	51.5	86.8	2.094	0.784	2.67	LLDA27813008791	LLNHPF712304013
	LL4/LM4	49.7	84.3	2.145	0.769	2.79	LLNHPF712246400	LLNHPF714116103

BW - body weight. DFI - daily feed intake. ADG - average daily gain over the assessed feeding period. FCR - feed conversion ratio. Half-sib: HL1 and LL1, HL2 and LL2; Full-sib: HL3 and LL3, HL4 and LL4.
