# Supplementary material for: Transcription Analysis of Liver and Muscle Tissues from Landrace Finishing Pigs with Different Feed Conversion Ratios
Source: Genes (Basel). 2022 Nov 8;13(11):2067. doi: 10.3390/genes13112067 (PMC9690258; doi:10.3390/genes13112067)
Supplement: Supplementary file 1 [file genes-13-02067-s001.zip › genes-2013343-SI/Supplementary Table S2.pdf]

Supplementary Table S2 Forward and reverse primers used for qPCR validation from RNASeq.

Target	Accession number	Sense and Antisense primer	PCR product (bp)
GAPDH	NC_010447.5	F:5'ACTTCGGCATCGTGGAG3'	128
		R:5' AGCAGGGATGATGTTCTGG3'	52
TRIM26	NC_010449.5	F:5' TCTCCTCCTCTGGCATCTG 3'	135
		R:5' GTTCTTGCGACTCTGCGTT 3'	52
ACAA2	NC_038254.2	F :5'CCGAGGTGTGTTCATCG3'	100
		R:5'GGCAGCAAATTCAGTCAAA3'	72
ANGPTL4	NC_010444.4	F:5'AGCGTCCCGAAATCCTC3'	175
		R:5' CACATTCACCTCGTCCCA3'	55
HP	NC_010448.4	F :5' CCAGAGAAGAAGACACCCA3'	134
		R:5' TCGTGAACAGCAAAGGC 3'	52
CTGF	NC_010443.5	F:5GGGATTGTATGCTGTGTGG3'	147
		R:5' GTCATTGGTGCTAAAGGCA3'	47
APOE	NC_010448.4	F :5'CTCTGGTGGAAGACATGCG3'	154
		R:5' GAGTTGGGCAGGAACGG3'	64
