# Supplementary material for: Transcription Analysis of Liver and Muscle Tissues from Landrace Finishing Pigs with Different Feed Conversion Ratios
Source: Genes (Basel). 2022 Nov 8;13(11):2067. doi: 10.3390/genes13112067 (PMC9690258; doi:10.3390/genes13112067)
Supplement: Supplementary file 1 [file genes-13-02067-s001.zip › genes-2013343-SI/Supplementary Table S3.pdf]

Supplementary Table S3. Transcriptome sequencing data of liver and muscle tissues of high and low feed efficiency.

sample	Q20（%）	Q30（%）	GC Count（%）	Total reads（million）	Unique map（%）	Multi map（%）	exon（%）	intron（%）	intergenic（%）
LL1	97.8	93.79	50.66	46.88	94.12	2.63	94.16	2.72	3.12
LL2	97.62	93.36	46.41	52.38	92.88	2.67	95.14	2.08	2.78
LL3	97.54	93	44.35	42.41	92.74	3.01	95.57	2.10	2.33
LL4	97.68	93.46	46.64	48.67	93.40	2.99	94.13	2.9	2.97
HL1	97.67	93.5	49.52	46.03	93.88	2.67	94.64	2.04	3.32
HL2	97.6	93.24	44.59	39.98	91.46	3.09	95.15	2.26	2.58
HL3	97.58	93.25	44.15	45.16	91.45	2.48	94.13	3.06	2.81
HL4	97.41	92.91	43.99	46.67	91.94	2.03	94.38	2.99	2.63
LM1	97.77	93.81	54.06	47.05	92.38	3.63	94.63	2.68	2.69
LM2	97.51	93.19	54.17	44.68	92.39	3.57	94.94	2.24	2.82
LM3	97.75	93.76	53.46	44.94	92.20	3.69	93.36	3.61	3.03
LM4	97.74	93.79	54.44	47.86	91.60	4.01	95.18	2.18	2.63
HM1	97.9	94.06	53.59	47.05	92.96	3.42	94.18	2.82	3.00
HM2	97.81	93.87	53.86	41.52	92.76	3.45	93.90	3.00	3.10
HM3	97.59	93.43	54.27	44.04	92.35	3.55	94.02	3.01	2.98
HM4	97.71	93.69	53.56	45.56	92.29	3.66	93.83	2.92	3.2

Q20, the ratio of Phred >20 bases; Q30, the ratio of Phred >30 bases; GC Count, the ratio of GC to the total bases; Total reads, the total number of sequences; Unique map, the unique position alignment rate; Multi map, multiple Position alignment rate; exon, exon; intron, intron ratio; intergenic, intergenic region ratio.
