# Supplementary figures and images for: Transcription Analysis of Liver and Muscle Tissues from Landrace Finishing Pigs with Different Feed Conversion Ratios
Source: Genes (Basel). 2022 Nov 8;13(11):2067. doi: 10.3390/genes13112067 (PMC9690258; doi:10.3390/genes13112067)

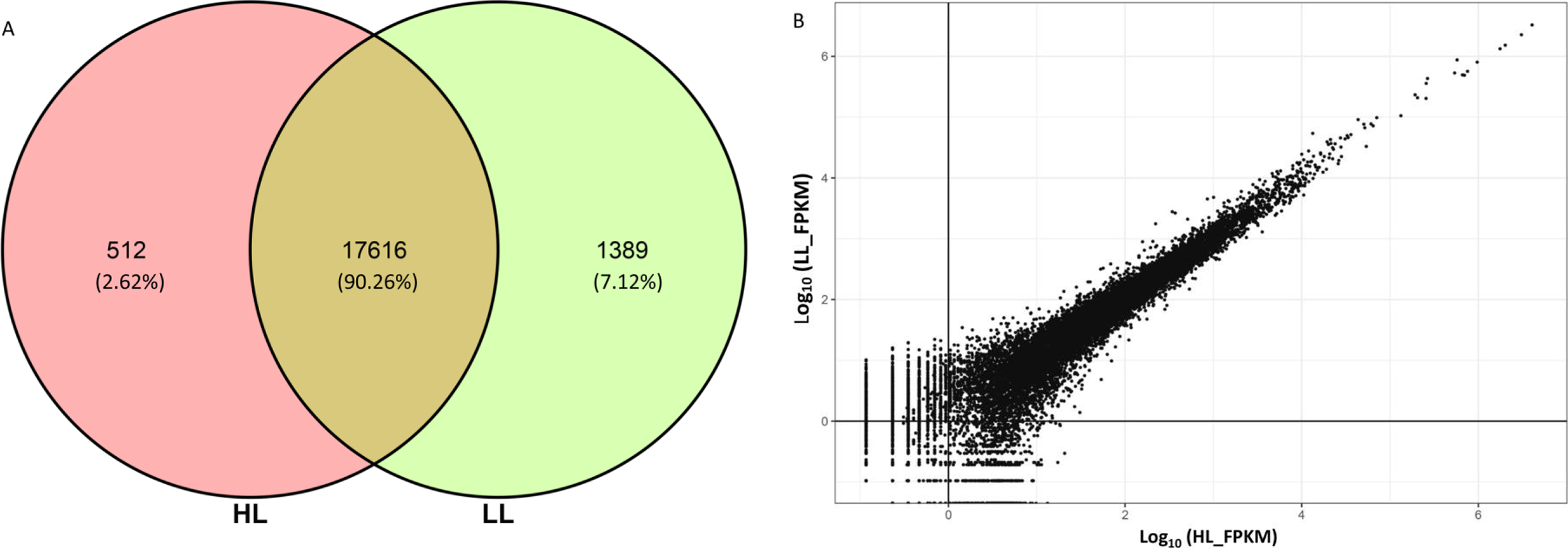

Supplement: Supplementary file 1 [file genes-13-02067-s001.zip › genes-2013343-SI/Supplementary Figure S1.tif]

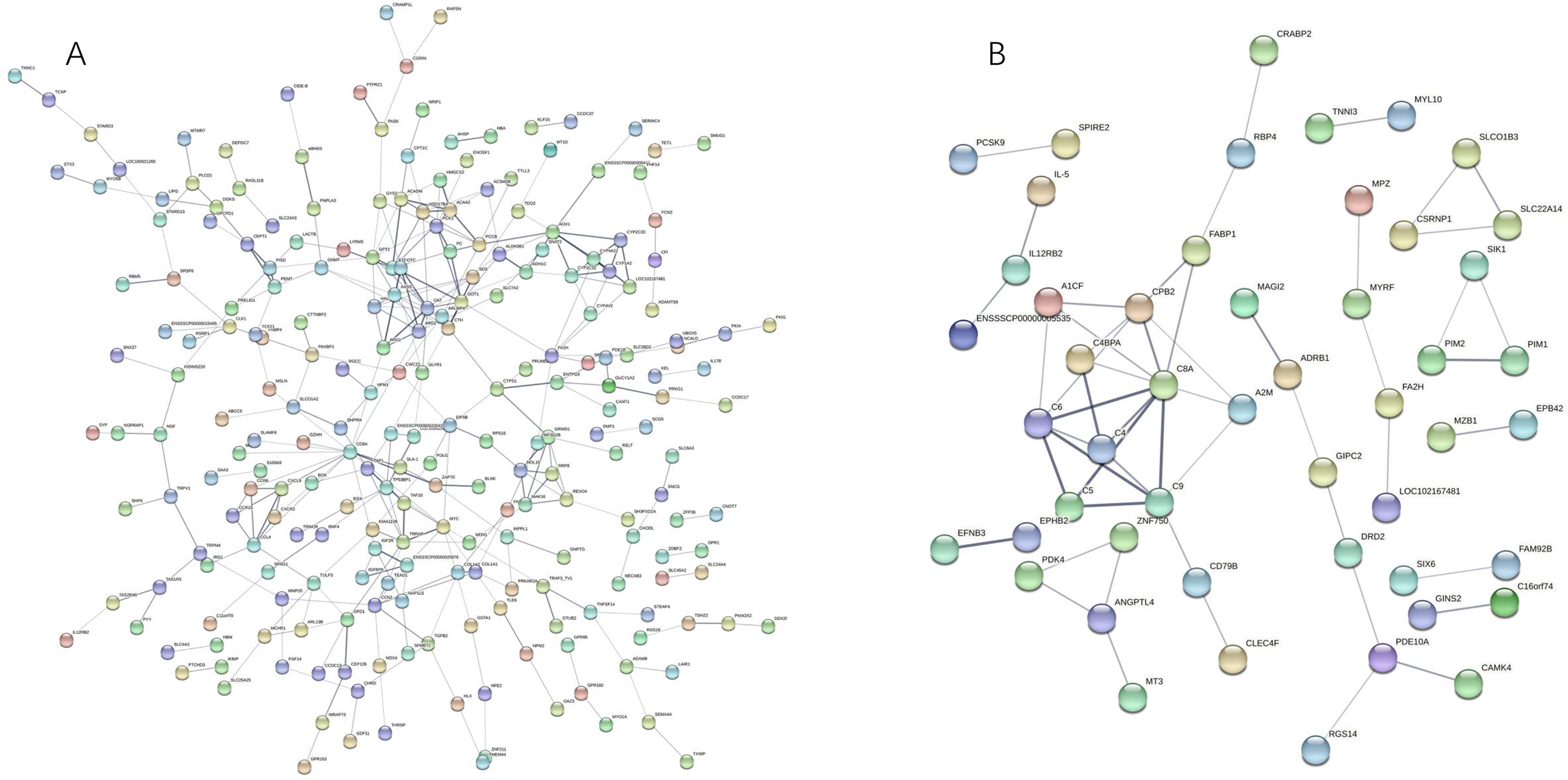

Supplement: Supplementary file 1 [file genes-13-02067-s001.zip › genes-2013343-SI/Supplementary Figure S2.tif]

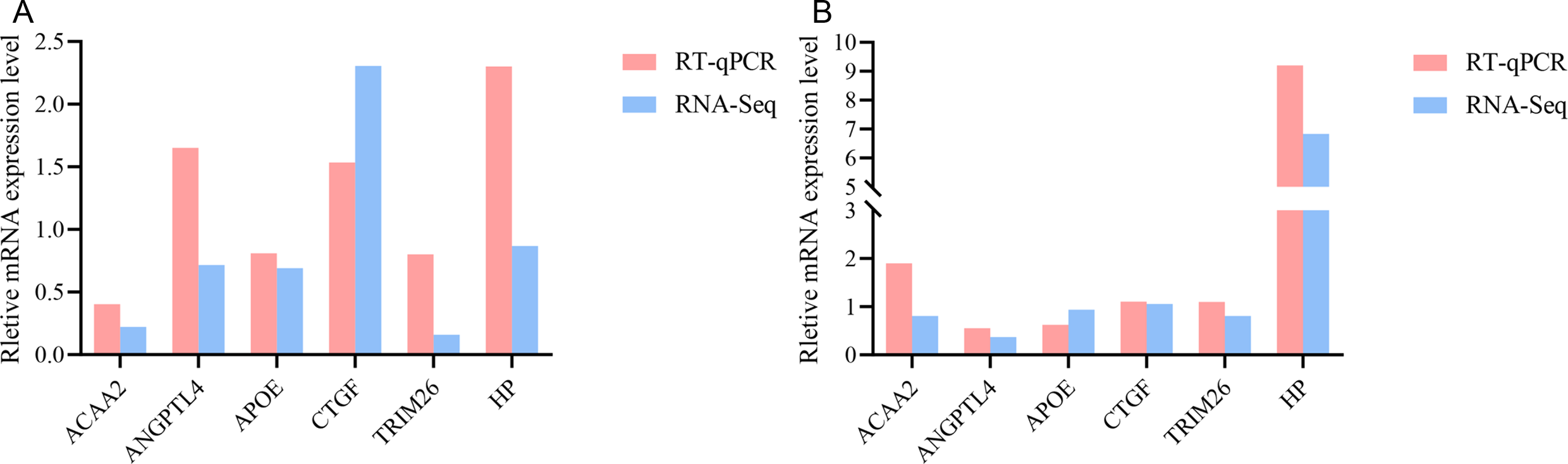

Supplement: Supplementary file 1 [file genes-13-02067-s001.zip › genes-2013343-SI/Supplementary Figure S3.tif]
